# Designing of Peptide Based Multi-Epitope Vaccine Construct against Gallbladder Cancer Using Immunoinformatics and Computational Approaches

**DOI:** 10.3390/vaccines10111850

**Published:** 2022-10-31

**Authors:** Mukhtar Ahmad Dar, Pawan Kumar, Prakash Kumar, Ashish Shrivastava, Muneer Ahmad Dar, Richa Chauhan, Vinita Trivedi, Ashutosh Singh, Eshan Khan, Ravichandiran Velayutham, Sameer Dhingra

**Affiliations:** 1 Department of Pharmacy Practice, National Institute of Pharmaceutical Education and Research (NIPER), Hajipur 844102, India; 2 Department of Biotechnology, National Institute of Pharmaceutical Education and Research (NIPER), Hajipur 844102, India; 3 Translational Bioinformatics and Computational Genomics Research Lab, Department of Life Sciences, Shiv Nadar University, G.B. Nagar 201314, India; 4 Division of Biotechnology, Sher-e-Kashmir University of Agricultural Sciences and Technology Kashmir (SKUAST-K), Shuhama 191201, India; 5 Department of Radiotherapy, Mahavir Cancer Sansthan and Research Centre (MCSRC), Phulwarisharif Patna 801505, India; 6 Department of Cancer Biology and Genetics, The Ohio State University, 460 West 12th Avenue, Columbus, OH 43210, USA; 7 Department of Natural Products, National Institute of Pharmaceutical Education and Research (NIPER), Kolkata 700054, India

**Keywords:** immunoinformatics, vaccine, epitope, antigenicity, TLR, GBC

## Abstract

Gallbladder cancer (GBC) is an aggressive and difficult to treat biliary tract carcinoma with a poor survival rate. The aim of this study was to design a peptide-based multi-epitope vaccine construct against GBC using immunoinformatics approaches. Three proteins implicated in the progression of GBC were selected for B and T cell epitope prediction and the designing of the potential vaccine construct. Seven CTL, four HTL and six Bcell epitopes along with a suitable adjuvant were selected and connected using linkers for designing the vaccine construct. The secondary and tertiary models of the designed vaccine were generated and satisfactorily validated. A Ramachandran plot of the final 3D model showed more than 90% of the residues in allowed regions and only 0.4% in disallowed regions. The binding affinity of a vaccine construct with TLR 2, 3 and 4 receptors was assessed through molecular docking and simulation. The average numbers of hydrogen bonds for vaccine-TLR 2, 3 and 4 complexes in the simulation were 15.36, 16.45, and 11.98, respectively, and remained consistent over a 100 ns simulation period, which is critical for their function. The results of this study provide a strong basis for further evaluation through in vitro/in vivo experimental validation of the safety and efficacy of the designed vaccine construct.

## 1. Introduction

The global burden of cancer is increasing rapidly with more than 19.3 million new incident cancers and 9.9 million deaths estimated in 2020 [1,2]. Worldwide, the incidence and mortality of GBC in 2020 was reported to be 115,949 and 84,695, respectively [1]. GBC is the most frequently diagnosed biliary tract cancer with poor prognosis and a high fatality rate [3]. The median survival of GBC is less than one year with an overall five-year survival rate ranging from 5–20% [4,5,6]. The poor survival of GBC is mostly associated with asymptomatic early stages leading to late-stage diagnosis on clinical presentation. More than 90% of GBC are diagnosed in advanced stages or the metastatic phase [7,8].

The incidence of GBC varies with geographic locations and other concomitant risk factors. The world age-standardized incidence and mortality rates (ASR) of GBC were 1.2 and 0.84, respectively. Data from Global Cancer Observatory-2020 suggests the highest incidence associated with GBC in Asia (70.8%), followed by Europe (10.8%), South America (6.8%), Africa (4.7%) and Northern America (4.5%) [2,9]. GBC is highly prevalent in India, Pakistan, Japan, Korea, Chile, Ecuador, Bolivia, Czech Republic, Poland and Slovakia [10].

Studies have identified cholelithiasis, cholecystitis, female gender and Salmonella infection as common risk factors associated with the development of GBC [5,11,12,13]. The ASR incidence in females and males is 1.8 and 0.97 respectively [9]. However, the association of different risk factors with the prognosis of GBC is poorly understood.

Asymptomatic progression and a lack of specific biomarkers for early detection leads to advanced stage presentation with aggressive tumor biology. Surgical resection of the gallbladder is recognized as the best treatment plan for GBC [14,15]. However, following curative surgery, the recurrence rate is high (35%) and prognosis remains poor, particularly in advanced disease [16]. Apart from surgery, chemotherapy with gemcitabine and oxaliplatin or gemcitabine and cisplatin are the mainstay of the treatment [17]. The benefits of adjuvant radiation therapy in GBC are unclear due to a lack of evidence regarding clinical outcomes and overall survival [17]. Conclusively, neither radical surgery nor chemotherapy have proved to be effective.

The overall prognosis and survival rates of most common cancers have improved as a result of advances in drug development, diagnostics and screening technologies. However, because GBC is an uncommon malignancy, research into its diagnosis and therapeutics is very limited. Malignant cells evade immune recognition by escaping T lymphocytes and natural killer cells and exploit the immunosuppressive tumor microenvironment [18]. Immunotherapies have lately received much traction as promising cancer therapeutic approaches. Vaccines stimulating the immune system to recognize and eliminate the cancer cells represents a novel and effective therapeutic approach [19,20,21].

Advanced immunoinformatics has revolutionized the field of immunological research and vaccine development [22]. A multi-epitope-based vaccine development strategy is a progressive approach for targeting cancer. Recently, EMD640744, an epitope-based vaccine, has moved to phase-1 clinical trials for solid tumors [23]. Immunoinformatics approaches have also been used for the development of potential vaccine constructs against SARS-CoV-2 [24,25] and other highly contagious viruses such as novovirus [26], mayarovirus [27] and Epstein-Barr virus [28]. With the advancements in immunology and bioinformatics, the immunoinformatics approaches provide an opportunity to develop specific, cost-effective and personalized biologics including vaccines [29].

As immune response plays a crucial role in fighting cancer, immunoinformatics approaches allow for the identification of the potential immunogenic T and B cell epitopes to trigger the desired immune response [23,30]. Epitope vaccines, particularly peptide-based multi-epitope vaccines, provide a uniquely designed, cost- and time-effective therapeutic strategy capable of inducing simultaneous humoral and cellular immune responses [31,32].

Against this backdrop, the discovery of novel alternative therapeutic interventions for GBC assumes critical importance. Researchers have identified 5′-nucleotidase isoform 2 (NT5E), aminopeptidase N (ANPEP) and membrane metallo-endopeptidase (MME) as potential targets for the diagnosis and treatment of GBC [19,20,33].

NT5E, also referred to as CD73 and coded by the NTSE gene, is a surface enzyme on B and T lymphocytes in humans that converts adenosine monophosphate (AMP) into adenosine [34]. Over-expression of NT5E leads to the accumulation of free adenosine which is negatively correlated with survival period in GBC [34,35]. The accumulating adenosine suppresses the cellular immune responses of regulatory T cells and tumor-associated macrophages, natural killer T (NKT) cells and helper T (Th1) cells, allowing malignant cells to evade detection thus promoting tumor growth and metastasis [36,37,38,39,40].

ANPEP, also referred to as CD13, is a membrane-bound zinc metallo-enzyme expressed in macrophages and other human cells [41]. Over-expression of ANPEP has been linked with tumor size, differentiation and metastasis in pancreatic, gastric, breast and gallbladder cancers [36,42]. Sanz et al. identified ANPEP activity in tumor tissue and plasma as an independent predictor and prognostic factor of 5-year survival in patients with colorectal cancer [41]. Being a ubiquitous enzyme with moonlighting functional roles limits its use as a potential therapeutic target. However, the latest evidence suggests a difference in the function and activity of ANPEP from cancer and normal cells [43].

Membrane metallo-endopeptidase (MME) or neprilysin is also a membrane-bound zinc metalloproteinase implicated in promoting cancer progression [36]. MME over-expression has been reported in breast, lung, hepatocellular and gallbladder cancers and has been associated with poor prognosis [44].

The identified proteins were used for the prediction of immunogenic epitopes and this study was aimed at designing a novel peptide-based multi-epitope vaccine construct against GBC using reverse vaccinology and immunoinformatics approaches. To our knowledge, this is the first study using these tools for designing such a peptide vaccine candidate targeting GBC.

## 2. Materials and Methods

### 2.1. Identification of Target Proteins and Sequence Retrieval

Based on their role in GBC; NT5E, ANPEP and MME proteins were selected for epitope prediction and the designing of a potential vaccine candidate. The sequences of the selected proteins in FASTA format were retrieved from the UniProtKB database for subsequent analysis. Their physicochemical properties, including stability and hydropathicity, were analyzed in the Protparam server [45]. The flow diagram in Supplementary Figure S1 depicts the process of vaccine construct development.

### 2.2. Prediction of Epitopes from Target Proteins

#### 2.2.1. Prediction, Screening and Selection of Cytotoxic T-Lymphocyte (CTL) Epitopes

CTL epitopes were predicted using the Immune Epitope Database (IEDB) MHC-I binding server [46]. An artificial neural network method with an HLA allele reference set was used for CTL prediction to cover most of the population [47,48]. The predicted CTL epitopes were ranked based on IC50 value, where a lower IC50 indicates a higher MHCI-I binding affinity. An IC50 of <50 nM indicates high affinity, <500 nM; moderate affinity and <5000 nM is considered a low affinity peptide. For this study, IC50 of <500 nM was used as a cut-off for CTL epitope prediction and selection for the vaccine construct. The predicted epitopes with IC50 of <500 nM were checked for immunogenicity, antigenicity, allergenicity and toxicity [46,49].

#### 2.2.2. Prediction, Screening and Selection of Helper T-Lymphocyte (HTL) Epitopes

HTL epitopes were predicted using the IEDB-MHCII server [50]. The NN-align 2.3 technique was selected as the prediction method and human HLA-DR was chosen as the locus [51,52]. The HTL epitopes with 15-mer length were retrieved and ranked on the basis of IC50 value with a lower IC50 indicating a higher MHCI-II binding affinity [53].

The predicted HTL epitopes with IC50 < 500 nM were evaluated for interferon-gamma (IFN-γ) inducing activity using the IFNepitope server. This server uses IFN-γ inducing and non-inducing datasets of MHC-II binders for the prediction and designing of HTL epitopes capable of generating an IFN-γ response [54]. The IFN-γ positive epitopes were further evaluated for antigenicity, immunogenicity, allergenicity and toxicity. Finally, immunogenic, antigenic, non-allergic, non-toxic and IFN-γ positive HTL epitopes were selected.

#### 2.2.3. MHC-I and MHC-II Population Coverage

The IEDB population coverage server was used to examine the selected CTL and HTL epitopes corresponding to MHC I and II families and binding leukocyte antigens [55]. The server computes the distribution and proportion of the population anticipated to respond to the selected epitopes. Moreover, the server also calculates the total numbers of epitope hits recognized by the total population. Population coverage was examined for world population and subcontinents, as well as countries such as India, Japan, South Korea and Chile, among others.

#### 2.2.4. Prediction, Screening and Selection of Linear B-Cell Epitopes

B-cell epitopes were predicted using ABCpred and BCPred servers. The ABCpred web server uses a recurrent neural network for epitope prediction [56]. The BCPred server prediction is based on a support vector machine algorithm [57]. The epitope length in BCPred was set at 16-mer in both servers using an overlap filter. Epitopes with a predicted score of ≤0.9 in BCPred and >0.5 in ABCpred were further evaluated for antigenicity, allergenicity and toxicity properties.

#### 2.2.5. Prediction of Antigenic, Immunogenic, Allergic and Toxic Properties

Antigenicity of the predicted epitopes was examined using the VaxiJen v.2.0 server with the target set to a tumor and antigenicity threshold of 0.5. This server uses an alignment-independent technique for antigenicity prediction based on the physicochemical parameters [58]. The immunogenicity of the T cell epitopes was examined using IEDB Class-I and Class-II immunogenicity servers [59,60].

The allergenicity of the predicted epitopes was analyzed using the AllerTOP v. 2.0 server. AllerTOP uses an auto cross covariance transformation algorithm and physicochemical characteristics of the proteins for allergenicity prediction. The server classifies epitopes as probable allergens or probable non-allergens on the basis of the k-nearest neighbor algorithm [61].

Each predicted epitope was subjected to in-silico toxicity analysis using the ToxinPred web server. ToxinPred is used for the prediction and design of non-toxic proteins/peptides [62].

#### 2.2.6. Designing of Vaccine Construct

Top scoring epitopes were selected and linked together for designing the final vaccine construct. The GPGPG linkers were added to link B-cell and HTL epitopes, whereas AAY linkers were used to connect CTL epitopes. To enhance the immunogenicity of the vaccine construct, human β-defensin 3 (hBD3) was used as an adjuvant. The adjuvant was connected to the construct using an EAAAK linker.

#### 2.2.7. Evaluation of Construct Physicochemical Properties

The physicochemical parameters were examined using the ProtParam tool [45]. These parameters included molecular weight, isoelectric point, atomic composition, stability, and hydropathicity. The solubility was predicted using the Protein-Sol web server [63]. The expected solubility value of >0.45 was estimated to have a higher solubility than the experimental dataset [64].

#### 2.2.8. Prediction of Secondary Structure

The PSIPRED server was used to assess the secondary structure of the designed vaccine sequence [65]. PSIPRED uses two stage neural networks to predict the 2D structure including α-helix, β-pleated sheets and coils [66].

#### 2.2.9. Prediction of Tertiary Structure

The RaptorX server was used to perform the homology modelling for the prediction of the tertiary structure [67]. The server is a template-based modelling tool for predicting 3D models and assigns a rank to the predicted models on the basis of a root mean square deviation (RMSD) score. The template-based threading and alignment quality predictions are the key components of RaptorX [68].

#### 2.2.10. Tertiary Structure Refinement and Validation

The template-based 3D model generated in the RaptorX was further refined using the Galaxy Refine web server [69]. The refinement helps to improve the quality of the 3D model. Galaxy Refine uses molecular dynamics simulation to achieve recurrent structure perturbation and overall structural relaxation through side chain repacking [70].

The quality of the refined 3D model was validated using the ERRAT and PROCHECK tools in the SAVES v6.0 web server [71,72,73,74,75]. ERRAT analyzes the overall quality of the 3D model and PROCHECK verifies the stereo-chemical quality by generating a Ramachandran plot of protein residues [71,74]. The validation of the final 3D model was further verified through the ProSA web server which compares the predicted model with known structures of proteins using NMR spectroscopy and X-ray analysis [76].

#### 2.2.11. Prediction of Discontinuous B-Cell Epitopes

The discontinuous B-cell epitopes of the validated 3D model were predicted using the ElliPro server [77]. The server predicts the surface-accessible nearby cluster residues based on their protrusion index (PI) values. This score is calculated by taking the average PI value of each residue. ElliPro came out on top when compared to other structure-based techniques for predicting epitopes, with an AUC value of 0.732 [77,78].

#### 2.2.12. Molecular Docking with Immune Receptors

The interaction between the designed construct and TLRs is crucial for immune response generation. TLR2, TLR3 and TLR4 were selected for docking purposes. The vaccine–receptor docking was performed using the HDOCK server to examine the binding affinity/energy of the docked complex [79]. The server uses a hybrid algorithm for protein–protein docking methodologies [80]. HDOCK provides a total of 100 docked complex predictions ranked on the basis of docked energy scores and ligand RMSD.

The vaccine–receptor interactions were visualized with the LigPlot+ v.2.2 server. LigPlot+ generates schematic 2D vaccine-receptor interaction diagrams representing intermolecular hydrogen bonds and hydrophobic interactions [81].

#### 2.2.13. Molecular Dynamics Simulation of Vaccine-Receptor Complexes

The stability and strength of the docked complexes were examined through molecular simulation [82]. The simulation was performed for each vaccine receptor docked complex for 100 ns using Desmond’s System builder panel with OPLS_2005 force field [83]. Before running the MD simulation, the system was equilibrated using the relax model system protocol. Ligand–receptor complexes were prepared by solvating with TIP3P water molecules, periodic boundary conditions established as a cubic box using buffer technique and a distance threshold of 10. The dynamics panel was set to default parameters, the trajectory was saved every 100 ps and the Like Energy was captured at 1.2 ps. At a temperature of 300 K and 1.01325 pressure, the volume of the box was equilibrated with the NPT ensemble (pressure).

The Noose–Hoover chain temperature coupling with 1.0 ps relaxation time and Martyna–Tobias–Klein pressure coupling 2.0 ps relaxation time of isotropic style were used during the simulation run at a 2 (fs) time step. The short-range method’s cut-off for columbic interaction was 9.0 radius.

#### 2.2.14. Immune Simulation

The generation of immunological response of the designed vaccine was demonstrated through immune simulation using the C-ImmSim server [84]. The prediction of immune response is based on the stimulation of three major anatomical systems including lymph node, thymus and bone marrow in mammals. Three injections were administered at 1, 84 and 168 time-steps (each time step corresponds to 8 h indicating that the vaccine was administered at intervals of 0, 28, and 56 days). The simulation volume was set at 50 and total simulation time was 1050 time-steps (350 days) and other parameters were kept as default. The output of the C-ImmSim server provides the simulation of different immunological cells and predicts the humoral immune response through immunoglobulins, cytokines and interleukin production.

## 3. Results

### 3.1. Identification of Target Proteins and Sequence Retrieval

Sequences of selected proteins, including NT5E (Uniprot ID-P21589), ANPEP (Uniprot ID-P15144) and MME (Uniprot ID-P08473) in FASTA format, were retrieved from the UniProtKB database. The stability of the proteins was verified by using the ProtParam server. The instability index of NT5E, ANPEP and MME was found to be 32.59, 36.17 and 37.62 respectively, indicating that the selected proteins were stable. The physicochemical properties of the selected proteins were examined and are provided in Supplementary Table S1. The FASTA sequences were subsequently used for B and T-cell epitope prediction for vaccine construction.

### 3.2. Prediction of CTL and HTL Epitopes

The CTL and HTL epitopes were predicted and selected on the basis of IC50 (<500 nM), antigenicity (>0.5), immunogenicity, non-allergenicity and non-toxicity from NT5E, ANPEP and MME proteins vaccine development. The selected CTL epitopes covering different Human Leucocyte Antigen (HLA) super types are depicted in Table 1. The predicted HTL epitopes were also evaluated for IFN-γ inducing properties. The selected HTL epitopes and corresponding HLA-DR super types with IFN-γ score and antigenicity are shown in Table 2.

### 3.3. CTL and HTL Population Coverage Analysis

In order to develop a feasible vaccine candidate relevant to the global population, it is important to take into account the population coverage of the selected T-cell epitopes. The world population coverage of selected CTL and HTL epitopes was 93.78% and 81.81% respectively. The coverage of the individual CTL and HTL epitopes and their respective HLA genotypic frequency is summarized in Table 3. The HTL epitopes had higher total HLA hits (48) as compared to CTL epitopes (22).

**Figure 1:**
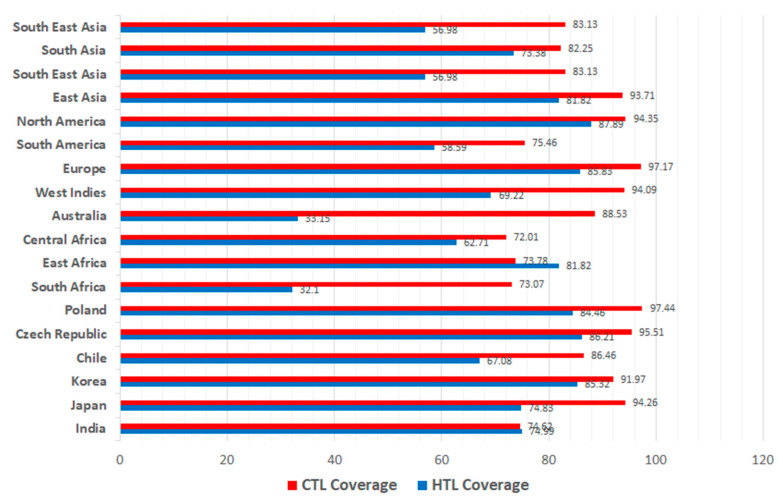
Population coverage of CTL and HTL epitopes with respect to different geographic regions.

The CTL and HTL population coverage was also assessed for different geographical regions such as South Asia (82.25%, 73.38%), South-East Asia (83.13%, 56.98), Europe (97.17%, 85.83%), South America (75.46, 58.59%), North America (94.35, 87.89%), East Africa (73.78%, 81.82%) and South Africa (78.07%, 32.10%). The coverage of CTL and HTL epitopes in different sub-continents and countries is shown in Figure 1.

### 3.4. Prediction of B-Cell Epitopes

The 16-mer B-cell epitopes were selected on the basis of binding score (>0.9). The epitopes with a high binding score were further evaluated for antigenicity (>0.5), allergenic and toxic properties. The antigenic, non-allergic and non-toxic B-cell epitopes were selected from three proteins as shown in Table 4.

### 3.5. Designing of Multi-Epitope Vaccine Construct

**Figure 2:**
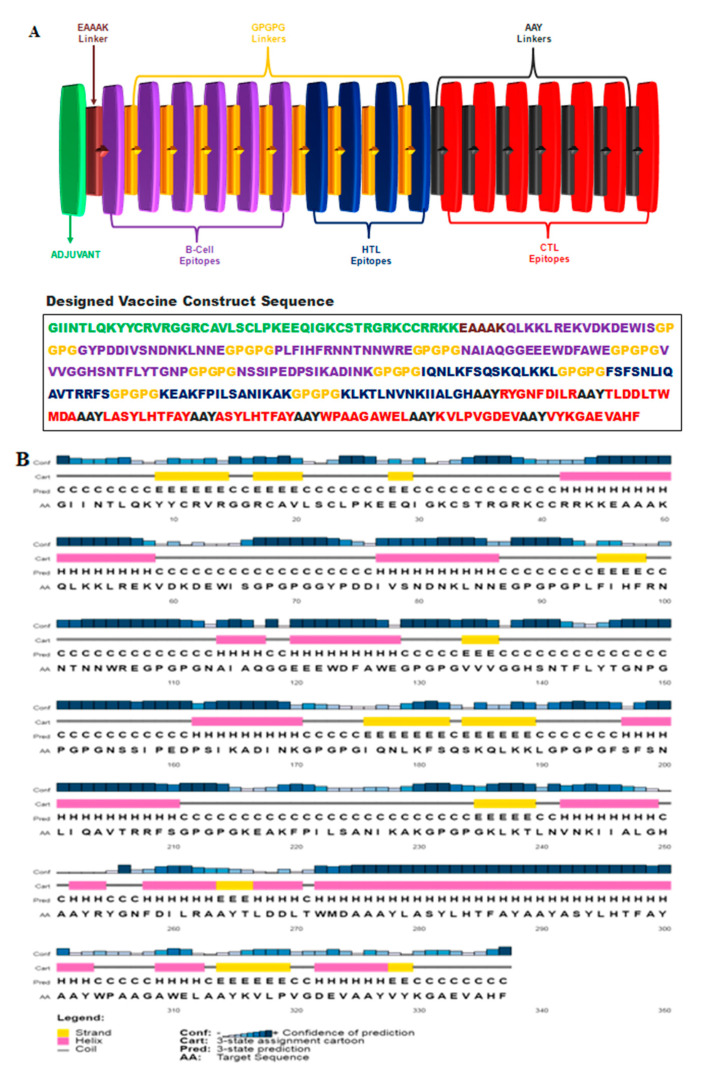
(**A**) Depicting designed multi-epitope vaccine construct and amino acid sequence. B cell and HTL epitopes were linked using GPGPG linkers, CTL epitopes were linked using AAY linkers and adjuvant was connected to N-terminal with EAAAK linker. (**B**) Predicted secondary structure of the epitope vaccine showing 37% alpha-helix, 14% beta-strand and 49% coils.

From the predicted epitopes, seven CTL, four HTL and six B-cell epitopes were selected for the design of the final vaccine construct. The GPGPG linkers were added to link B-cells and HTL epitopes, whereas AAY linkers were used to connect CTL epitopes. hBD3 (ID-Q5U7J2), a 45 amino acid long adjuvant, was added to improve the immunogenicity of the vaccine construct. The adjuvant was linked using EAAAK linkers. The schematic diagram and amino acid sequence of the designed vaccine construct is shown in Figure 2A.

The overall antigenicity score was found to be 0.71, indicating that the vaccine construct is highly antigenic and classified as a probable non-allergen.

### 3.6. Evaluation of Construct Physicochemical Properties

The final construct consisted of 337 amino acids with a molecular weight of 36.21 KDa (C_1644_H_2513_N_453_O_468_S_7_) and an isoelectric point of 9.28. The predicted aliphatic index was 70.47 suggesting thermo-stability and an instability index of 23.39 confirmed that the vaccine construct was stable. The GRAVY score was found to be −0.445, suggesting its hydrophilic nature. The estimated half-life was predicted to be 30 h in mammalian reticulocytes, >20 h in yeast and >10 h in *E. coli.*

### 3.7. Prediction of Secondary and Tertiary Structure

**Figure 3:**
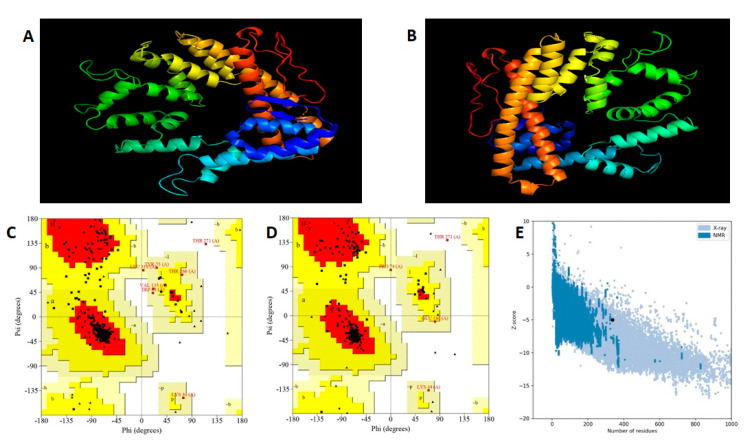
Vaccine construct 3D structure modelling, refinement and validation. (**A**) Tertiary structure of the vaccine construct using RaptorX. (**B**) Refined 3D model of the vaccine construct using Galaxy Refine. (**C**) Initial Ramachandran plot showing 87.2% residues in most favored regions, 12.5% residues in additional and generously allowed regions, and 0.4% residues in disallowed regions. (**D**) Ramachandran plot after refinement showing 92.1% in favor, 6.8% in allowed and 0.4% in disallowed regions of protein residues. (**E**) Z score of refined model using ProSA-web, showing a score of −5.2.

The secondary structure consisted of 37% alpha-helix, 14% beta-strand and 49% coils. The 2D structure is shown in Figure 2B. RaptorX generated five 3D models of the final vaccine sequence with RMSD values ranging from 4.563 to 7.726. Each model was evaluated for overall quality using ERRAT and PROCHECK tools. Model 2 with RMSD-5.2374 was selected on the basis of the overall quality factor in ERRAT (93.7%) and PROCHECK (Ramachandran plot). The Ramachandran plot of Model 2 showed 87.2% of amino acid residues in the most favored, 12.5% in allowed and 0.4% in disallowed regions. The initial 3D model and associated Ramachandran plot are shown in Figure 3A,C.

### 3.8. Tertiary Structure Refinement and Validation

Galaxy Refine generated five models of which Model-3, with an RMSD of 0.359, a Mol Probity of 1.965, a GDT-HA of 0.9733, a Clash score of 12.7 and poor rotamers of 0.4, was selected for further analysis.

The Ramachandran plot of the refined 3D model showed 92.1% residues in most favored, 6.8% in additional allowed regions, 0.8% in generously allowed regions and 0.4% in disallowed regions. Based on the results of the Ramachandran plot and ERRAT score, that quality of Model-3 was found to be acceptable. The refined 3D model and its Ramachandran plot are shown in Figure 3B,D respectively. The 3D model was further examined and validated through the ProSA Web server with a predicted Z-score of −5.2, being in the range of experimentally validated protein structures obtained from X-ray and NMR spectroscopy analysis (Figure 3E). The solubility score was found to be 0.523 which shows that the construct is soluble upon expression (Supplementary Figure S2).

### 3.9. Prediction of Discontinuous B-Cell Epitopes

**Figure 4:**
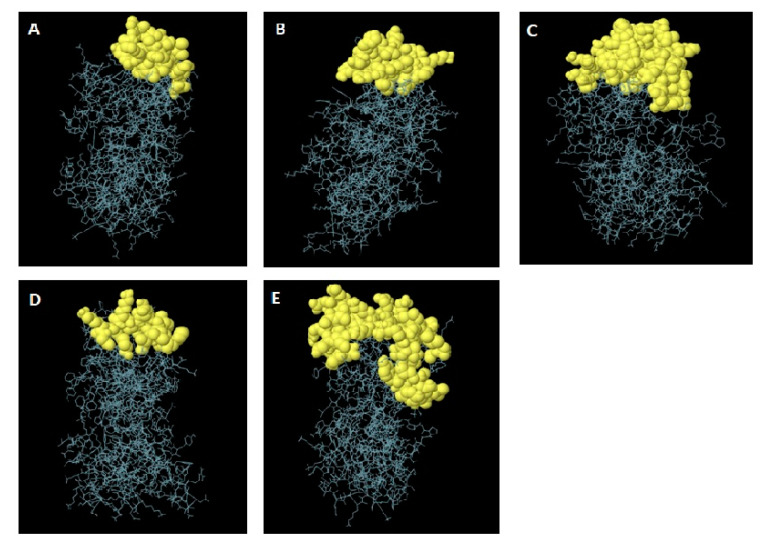
Discontinuous B cell epitopes in the 3D model of the designed construct. (**A**–**E**) The grey sticks represent the bulk of the peptide construct and the yellow surfaces depict the residues of discontinuous B cell epitopes.

A total of six discontinuous B-cell epitopes were identified by the ElliPro server. Almost five B-cell epitopes contained 167 amino acid residues present in the main region of the vaccine construct. A score value in the range of 0.67–0.76 was chosen for the selection of discontinuous B-cell epitopes (Figure 4). Twenty-two residues were predicted from 60–61, 63, 65–81 and 84–85. Twenty-four residues were predicted from 300–303, 305–314 and 328–337. Sixty residues were predicted from 97, 100–110, 121, 124, 125, 128–141, 143–145, 148–161, 182, 184, 185 and 187–197. Thirteen residues were predicted from 220–231 and 234. Forty-eight residues were predicted from 1–7, 9–11, 13–23, 27, 44–47, 49, 50, 53 and 256–274 (Supplementary Table S2).

### 3.10. Molecular Docking with Immune Receptors

**Figure 5:**
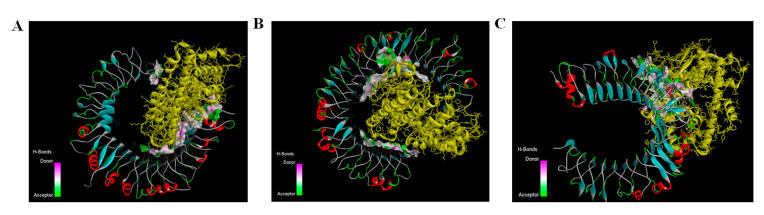
Molecular docking and 3D structures of vaccine–receptor complexes (**A**) Vaccine—TLR2 docked complex (**B**) Vaccine—TLR3 docked complex (**C**) Vaccine—TLR-4 docked complex.

**Figure 6:**
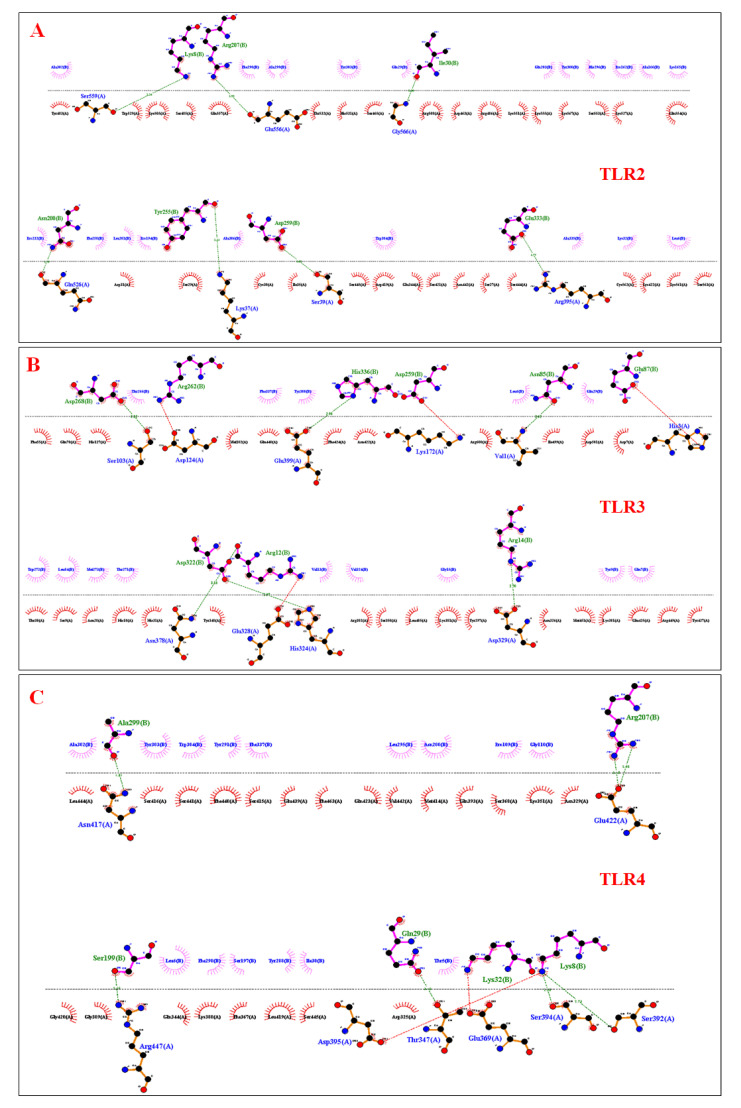
Intermolecular hydrogen bonds and hydrophobic interactions represented in 2D structures for TLRs and vaccine construct complex (**A**) Vaccine—TLR2 docked complex (**B**) Vaccine—TLR3 docked complex (**C**) Vaccine—TLR-4 docked complex.

The docking of the construct was performed with immune receptors including TLR2, TLR3 and TLR4. Three-dimensional (3D) docked scores for TLR-2, TLR-3 and TLR-4 were −344.38, −345.38 and −324.47 respectively. The docking energy scores indicating the vaccine–receptor complex binding affinity and ligand RMSD are shown in Supplementary Table S3 and the vaccine—TLR2, TLR3 and TLR4—docked complexes are shown in Figure 5A–C. The docked complexes were analyzed in LigPlot+ for visualization of intermolecular hydrogen bonds representing vaccine–receptor interactions. For the ligand-TLR2 complex, Lys8, Arg207, Ile30, Asn200, Tyr255, Asp259 and Glu333 residues were involved in intermolecular hydrogen bonding (Figure 6A). Similarly, Asp268, Arg262, His336, Asp259, Asn85, Glu87, Asp322, Arg12, Arg14 residues in ligand-TLR3 (Figure 6B) and Ala299, Arg207, Ser199, Gln29, Lys32, Lys8 residues in ligand-TLR4 (Figure 6C) were identified in LigPlot.

### 3.11. Molecular Dynamics Simulation of Receptor-Vaccine Complex

**Figure 7:**
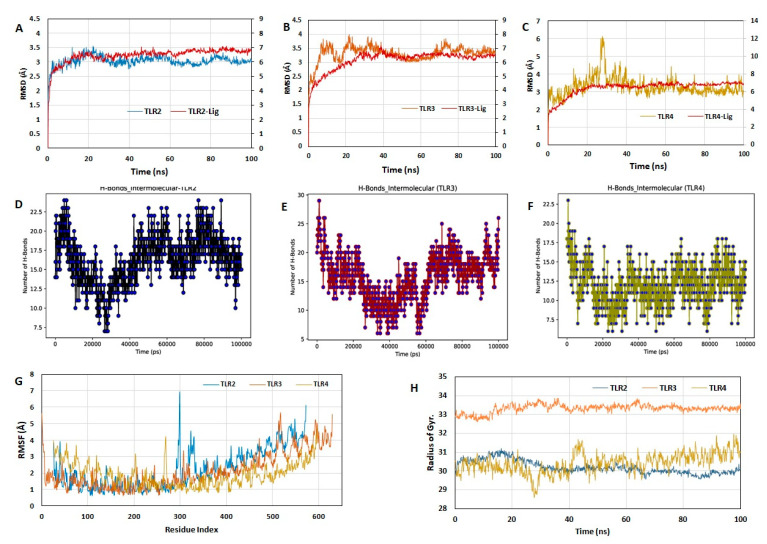
Molecular dynamics simulation of vaccine and immune receptors. (**A**–**C**) RMSD plot of docked vaccine construct with TLR2, TLR3 and TLR4 receptors respectively. (**D**–**F**) Intermolecular H-bonds of docked vaccine construct with TLR2, TLR3 and TLR4 receptors respectively. (**G**) RMSF plot of TLR2, TLR3 and TLR4 receptors. (**H**) Radius of gyration of TLR2, TLR3 and TLR4 receptors.

The Maestro’s Schrodinger simulation event analysis module was used to analyze the trajectories. By superimposing the trajectories over the reference frame, RMSD and RMSF were calculated using trajectories from MD simulation data. This provides an estimate of conformational stability and volatility over the course of the simulation. The amount of hydrogen bonds established between the receptor and the docked ligand during the simulation also suggests the ligand’s binding stability with the receptor. A binding arrangement with a higher number of hydrogen bonds is said to be more stable. For each TLR2, TLR3, and TLR4 receptor complex with the ligand, the RMSD was computed. TLR2-complex RMSD demonstrated that, after 60 ns, the RMSD of the TLR2 protein and the ligand converged. Throughout the simulation, the protein–ligand complex remained stable. For TLR2 and the TLR2-bound ligand, the standard deviations in RMSD were 0.932 and 1.4321, respectively, with average RMSD values of 2.75 and 5.59 (Figure 7A). For both the TLR3 protein and ligand, the RMSD plot of the TLR3-complex revealed convergence after 5 ns and remained stable throughout the simulation (Figure 7B). The RMSD of the ligand was raised to around 20–60 ns, but the binding site remained unchanged. TLR3 and the TLR3-bound ligand had standard deviations of 0.231 and 0.954, respectively, with average RMSD values of 3.07 and 5.75 (Figure 7B). The TLR4-complex RMSD data demonstrate convergence after 5 ns for both the TLR4 protein and the ligand. Ligand RMSD increased up to 20 ns before stabilizing towards the end of the simulation. Throughout the simulation, the TLR4-ligand complex maintained its conformational stability (Figure 7C). This shows that the protein–ligand complex was stable throughout the simulation. The standard deviation in RMSD was 0.314 Å and 0.885 Å, while average RMSD values were 3.07 Å and 6.03 Å, for TLR4 and the TLR4-bound ligand, respectively.

Throughout the simulation, the compactness of the proteins was examined, and the plot revealed that all three proteins were folded correctly. In comparison to TLR3 and TLR4, TLR2 demonstrated changes in compactness during the simulation. The contact between the two molecules is represented by intermolecular hydrogen bonds (H-Bonds), which are implicated in the intensity of binding through the number of H-Bonds. The more H-bonds there are, the more binding or interaction there is between two molecules. Throughout the simulation, the average number of intermolecular H-bonds between complex TLR2–ligand (Figure 7D), TLR3–ligand (Figure 7E), and TLR4–ligand (Figure 7F) were 15.36, 16.45, and 11.98 respectively. For complex TLR2–ligand, TLR3–ligand, and TLR4–ligand, the range of hydrogen bonds during 100 ns simulation over 1000 time frames was 7–24, 6–29, and 6–23, respectively. The number of hydrogen bond interactions at 10 ns time intervals with RMSD values is shown in Table 5. This MD simulation analysis indicates that all three immune receptors efficiently bound with the ligand and showed stable binding throughout the simulation (Supplementary Videos S1–S3). The number of hydrogen bonds in the 100 ns simulation over 1000 time frames and corresponding receptor and ligand RMSD are provided in Supplementary Table S4.

The average values of RMSF for TLR2, TLR3, and TLR4 were 1.23 Å, 2.71 Å, and 2.45 Å, respectively. RMSF showed fewer fluctuations in TLR3 and TLR4 as compared to TLR2, see Figure 7G. Moreover, the ligand’s RMSF plot indicates less fluctuation in the RMSF values of TLR2 as compared to TLR3- and TLR4-bound ligands. Figure 7H shows the radius of gyration of all three complexes.

### 3.12. Immune Simulations of Final Vaccine Construct

The output of the C-ImmSim server provides the simulation of different immunological cells including B-cell, helper-T cell (TH), cytotoxic-T cell (TC), natural killer cell (NK), macrophages (MA) and the dendritic cell (DC) population. Moreover, the prediction of immunoglobulins, cytokines and interleukin production during the immune simulation is also provided.

**Figure 8:**
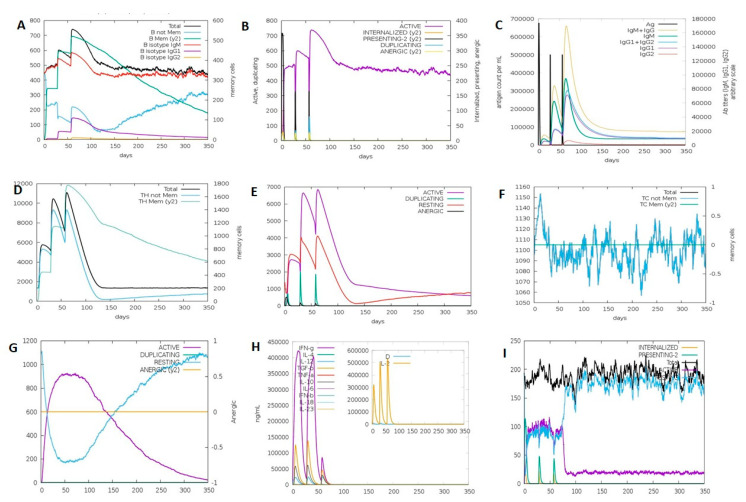
Demonstration of humoral and cellular immune responses using immune simulation. (**A**) B-cell population (cells per mm^3^). (**B**) B-cell population per state (cells per mm^3^). (**C**) Antigen, immunoglobulins and immunocomplexes. (**D**) TH cell population (cells per mm^3^). (**E**) TH cell population per state (cells per mm^3^). (**F**) TC cell population (cells per mm^3^). (**G**) TC cell population per state (cells per mm^3^). (**H**) Concentrations of cytokines and interleukins production. (**I**) Macrophage population per state (cells per mm^3^).

The total B-cell population, memory B-cell and active B-cell population increased following each booster vaccine dose and remained stable with minimal decay over the period of 350 days, see Figure 8A,B. There was a considerable rise in antibody response with every exposure to the vaccine construct with a corresponding decrease in the antigen levels. The humoral immune response was characterized by IgG and IgM antibodies and the IgM response was higher as compared to IgG after each vaccination (Figure 8C). The active TH cells and memory TH cells spiked after second and third vaccine doses which remained elevated up to 350 days (Figure 8D,E). Similarly, the TC cell population per state showed a steady increase in the active TC population (Figure 8F,G). The IFN-γ response was significantly higher after the first and second doses and the concentrations of IL-10 and TGF-b also spiked following each vaccination (Figure 8H). Throughout the simulation, there was also a concurrent rise in the activity of macrophages, dendritic and natural killer cells (Figure 8I).

## 4. Discussion

GBC is a biliary tract carcinoma with poor prognosis, high death rate, and a survival rate of <1 year. Gallbladder malignancies are highly aggressive and current treatments have yielded dismal outcomes [3,4,5,6]. Consequently, the development of novel innovative therapies for the improvement of survival in this patient population are urgently needed.

Priya et al. in a recent study reported significant over-expression in NT5E, ANPEP and MME proteins in GBC patients as compared to the control groups, highlighting their potential as diagnostic and therapeutic targets [36]. The authors reported that NT5E levels (expressed by cancer exosomes) were significantly elevated in the advanced stages of GBC; ANPEP was increased in early as well as later GBC while s MME was significantly higher in the early stages of GBC. These tumor-associated extracellular vesicular proteins are implicated in tumor progression and immune suppression [36]. Finally, these proteins were selected as potential targets for antigenic epitope prediction to stimulate the immune system and combat GBC progression.

Reverse vaccinology and immunoinformatics approaches in vaccine development is a rapidly developing field. The epitope-based vaccines designed using these approaches have demonstrated in vivo efficacy as well as protective immunity with several vaccines undergoing clinical trials [31]. Several studies have shown promising results in developing epitope vaccines against different cancers such as breast cancer, Kaposi sarcoma, colon cancer and cervical cancer [85,86,87,88]. Lately, peptide-based vaccines have gained traction due to several advantages as compared to the conventional vaccines. Apart from the capability of inducing cancer-specific immune responses, the peptide vaccines are relatively safe and incur lower developmental costs [89].

Cellular immunity is generated upon the binding of immunogenic peptides to MHC I and II. The selection of suitable epitopes capable of eliciting a good immune response with maximum population coverage is critical [90]. In this study, the CTL and HTL epitopes were selected through rigorous screening. Initially, predicted epitopes were ranked on the basis of IC50 and an IC50 value of <500 nM was used as a threshold. The predicted epitopes were further examined for immunogenicity, population coverage, antigenicity, IFN-γ inducing capability and non-allergenicity. Designed with both CD4+, CD8+ immunogenic epitopes, this epitope vaccine could elicit a strong, long-lasting cellular immunity.

Finally, the top seven CTL, four HTL and six B-cell predicted epitopes were linked using different linkers for the designing of a final vaccine construct. hBD3 was added as an adjuvant for improved immunogenicity. hBD3 acts as an immune regulator by stimulating monocytes and dendritic cells, thereby playing a crucial role in activating T cells and cytokine production [91].

The safety and efficacy of the vaccine is determined by the population in which it is to be administered. Representing both MHC I and II alleles, the maximum world population coverage was 93.78% for CTL epitopes and 81.81% for HTL epitopes, making it a promising vaccine candidate. The class I and class II epitopes showed an excellent population coverage in prevalent geographic regions such as India (74.02% & 74.99%), Japan (94.26% & 74.83%), Korea (91.97% & 85.32%) and Chile (86.46% & 67.08%), among others.

Finally, a 337 amino acids vaccine construct was designed and evaluated for stability, antigenicity, and physicochemical properties. The construct was stable as indicated by the predicted aliphatic index and instability index. Additionally, the construct also demonstrated a higher solubility than the soluble *E. coli* proteins from the experimental dataset [63,64]. Antigenicity and allergenicity are critical factors in multi-epitope vaccine development and because these properties were assessed before designing the construct, the final vaccine was found to be highly antigenic (0.71), non-allergic and non-toxic in nature.

The secondary and tertiary models of the vaccine were generated and satisfactorily validated. A Ramachandran plot of the final 3D model showed more than 90% of the residues in allowed regions and only 0.4% in disallowed regions. For successful generation of immune response, the stimulation of immunological receptors such as TLRs is important. Activation of TLRs in immune and cancer cells is critical in triggering cancer-associated immune response through multiple signaling pathways [92]. The binding affinity of the designed construct with TLR2, TLR3 and TLR4 was predicted using molecular docking and the stability of the vaccine–receptor docked complexes was examined through stability, hydrogen bonds and simulation trajectories using molecular dynamics simulation. According to molecular docking experiments, the vaccine has a strong affinity for TLR-2, TLR-3, and TLR-4 receptors. The average numbers of hydrogen bonds for vaccine-TLR 2, 3 and 4 complexes were 15.36, 16.45, and 11.98, respectively, and remained consistent over a 100 ns simulation period, which is critical for their function.

The developed vaccine candidate demonstrated an acceptable cellular as well as humoral immune response in the immune simulation study [84]. Since the vaccine contained both CTL and HTL, it showed stimulation of the respective immune cells, which may further lead to the activation of other potential immune cells such as NK cells, macrophages and dendritic cells via complex signaling. Overall, the results of immune simulation showed that the immune response increased in tandem with each booster dose, corresponding to the activation of multiple immune cells. Moreover, the vaccine construct containing several linear and discontinuous B-cell epitopes suggested antibody mediated immune response properties which were clearly seen as increased levels of IgG and IgM during immune simulation.

A major limitation of this study is the lack of the experimental validation and evaluation of the safety and efficacy of the designed vaccine construct. However, the results of this study provide a strong basis for further in vitro/in vivo studies to demonstrate the safety and efficacy of the construct.

## 5. Conclusions

Stimulation of the immune system is critical in combating cancer, and peptide-based epitope vaccines have demonstrated the capability of generating a cancer-specific immune response. This study reports the designing of a peptide-based multi-epitope vaccine construct with a thorough analysis of its immunogenicity, antigenicity, allergenicity and stability using immunoinformatics approaches to trigger a robust immune response against GBC. The construct contains CD4+, CD8+ and B-cell epitopes from three different antigenic proteins implicated in GBC progression. The interaction and binding strength of this vaccine construct with TLRs was excellent and the in silico immune simulation has shown its ability to induce both cellular and antibody mediated immune responses. The promising results in the present study provide a strong basis for further evaluation through in vitro and in vivo experimental validation of the safety and efficacy of the designed vaccine candidate.

## Figures and Tables

**Table 1:** List of CTL epitopes selected for design of vaccine construct.

CTL Epitope	Sequence	Start	End	Length	Alleles	IC-50	Rank	Antigenicity/ Immunogenicity	Allergenicity/Toxicity
**MME Protein**									
RYGNFDILR	2	43	51	9	HLA-A*31:01	8.38	0.06	1.49 and 0.24	NA/NT *
	2	43	51	9	HLA-A*33:01	454.92	1.4		
TLDDLTWMDA	8	34	43	10	HLA-A*02:01	54.65	0.5	0.85 and 0.12	NA/NT
	8	34	43	10	HLA-A*02:06	196.1	1.5		
**ANPEP Protein**									
LASYLHTFAY	9	20	29	10	HLA-B*35:01	11.92	0.04	0.99 and 0.12	NA/NT
	9	20	29	10	HLA-B*15:01	69.78	0.34		
	9	20	29	10	HLA-A*30:02	105.04	0.29		
	9	20	29	10	HLA-A*01:01	125.1	0.2		
ASYLHTFAY	9	21	29	9	HLA-A*11:01	19.95	0.1	0.9536 and 0.17	NA/NT
	9	21	29	9	HLA-A*30:02	31.44	0.06		
	9	21	29	9	HLA-B*35:01	32.57	0.1		
	9	21	29	9	HLA-A*03:01	63.6	0.25		
	9	21	29	9	HLA-A*01:01	81.21	0.15		
	9	21	29	9	HLA-A*32:01	108.79	0.1		
	9	21	29	9	HLA-B*15:01	129.05	0.55		
**NT5E Protein**									
WPAAGAWEL	1	21	29	9	HLA-B*35:01	7.79	0.02	0.50 and 0.36	NA/NT
	1	21	29	9	HLA-B*07:02	35.07	0.14		
	1	21	29	9	HLA-B*53:01	235.07	0.18		
KVLPVGDEV	3	42	50	9	HLA-A*02:06	14.43	0.15	1.1955 and 0.13	NA/NT
	3	42	50	9	HLA-A*02:01	286.68	2		
VYKGAEVAHF	2	35	44	10	HLA-A*23:01	23.91	0.08	1.1216 and 0.19	NA/NT
	2	35	44	10	HLA-A*24:02	65.7	0.12		

* NA: Non-Allergic; NT: Non-Toxic.

**Table 2:** List of HTL epitopes selected for design of vaccine construct.

HTL PEPTIDE	Seq no	Start	End	Length	ALLELE	IC50	IFN-γ Score	Antigenicity/Immunogenicity	Allergenicity/Toxicity
**MME Protein**									
IQNLKFSQSKQLKKL	9	32	46	15	HLA-DRB1*07:01	5.5	Positive	0.84/92.7	NA/NT *
	9	32	46	15	HLA-DRB5*01:01	20.7			
	9	32	46	15	HLA-DRB1*01:01	27.1			
	9	32	46	15	HLA-DRB1*09:01	41.3			
	9	32	46	15	HLA-DRB1*13:02	61.8			
	9	32	46	15	HLA-DRB1*15:01	152.7			
	9	32	46	15	HLA-DRB1*11:01	218.6			
	9	32	46	15	HLA-DRB1*08:02	379.8			
	9	32	46	15	HLA-DRB4*01:01	412.6			
	9	32	46	15	HLA-DRB1*03:01	458.6			
	9	32	46	15	HLA-DRB3*02:02	491.9			
	5	5	19	15	HLA-DPA1*03:01/DPB1*04:02	105.8			
	5	5	19	15	HLA-DPA1*01:03/DPB1*04:01	302.3			
	5	5	19	15	HLA-DQA1*04:01/DQB1*04:02	432.7			
	5	5	19	15	HLA-DPA1*01:03/DPB1*02:01	438			
	5	5	19	15	HLA-DPA1*02:01/DPB1*05:01	488.9			
**ANPEP Protein**									
FSFSNLIQAVTRRFS	1	896	910	15	HLA-DRB1*01:01	13.5	Positive	0.63/83.3	NA/NT
	1	896	910	15	HLA-DRB5*01:01	14.7			
	1	896	910	15	HLA-DRB1*11:01	16.6			
	1	896	910	15	HLA-DRB1*09:01	22.1			
	1	896	910	15	HLA-DRB1*07:01	34			
	1	896	910	15	HLA-DRB1*13:02	39.6			
	1	896	910	15	HLA-DRB1*15:01	74.6			
	1	896	910	15	HLA-DRB1*08:02	81.3			
	1	896	910	15	HLA-DRB1*04:01	101			
	1	896	910	15	HLA-DRB1*04:05	167			
	1	896	910	15	HLA-DRB3*02:02	175.3			
	1	896	910	15	HLA-DRB1*03:01	212.8			
	1	896	910	15	HLA-DRB3*01:01	360.9			
	1	896	910	15	HLA-DRB1*12:01	392.6			
**NT5E Protein**									
KEAKFPILSANIKAK	3	13	27	15	HLA-DRB5*01:01	24.7	Positive	0.70/86.7	NA/NT
	3	13	27	15	HLA-DRB1*01:01	39.6			
	3	13	27	15	HLA-DRB1*15:01	63.2			
	3	13	27	15	HLA-DRB4*01:01	73.8			
	3	13	27	15	HLA-DRB1*07:01	103.6			
	3	13	27	15	HLA-DRB1*13:02	144.2			
	3	13	27	15	HLA-DRB1*11:01	200.6			
	3	13	27	15	HLA-DRB1*09:01	270.7			
	3	13	27	15	HLA-DRB1*04:05	387.2			
	3	13	27	15	HLA-DRB1*08:02	407.4			
	3	13	27	15	HLA-DRB1*04:01	484.4			
	3	13	27	15	HLA-DPA1*02:01/DPB1*14:01	282.4			
	3	13	27	15	HLA-DPA1*02:01/DPB1*05:01	283.7			
	3	13	27	15	HLA-DPA1*03:01/DPB1*04:02	364.6			
KLKTLNVNKIIALGH	4	26	40	15	HLA-DRB1*13:02	6.3	Positive	1.0/69.3	NA/NT
	4	26	40	15	HLA-DRB3*02:02	21			
	4	26	40	15	HLA-DRB1*01:01	31.6			
	4	26	40	15	HLA-DRB1*07:01	71			
	4	26	40	15	HLA-DRB4*01:01	71.2			
	4	26	40	15	HLA-DRB1*11:01	75.6			
	4	26	40	15	HLA-DRB1*12:01	77.5			
	4	26	40	15	HLA-DRB5*01:01	80.6			
	4	26	40	15	HLA-DRB1*04:01	195.4			
	4	26	40	15	HLA-DRB1*08:02	318.5			
	4	26	40	15	HLA-DRB1*15:01	371.7			
	4	26	40	15	HLA-DRB1*03:01	478.1			
	4	26	40	15	HLA-DPA1*03:01/DPB1*04:02	121.1			

* NA: Non-Allergic; NT: Non-Toxic.

**Table 3:** World population coverage of individual CTL and HTL epitopes (MHC class-I and MHC class-II).

**EPITOPES**	**Coverage**	**HLA Allele (Genotypic Frequency (%)**															**Total HLA Hits**
	**Class I**	A*01:01 (10.09)	A*02:01 (24.39)	A*02:06 (1.09)	A*03:01 (9.77)	A*11:01 (8.99)	A*23:01 (3.06)	A*24:02 (12.59)	A*30:02 (1.36)	A*31:01 (3.02)	A*32:01 (2.59)	A*33:01 (0.99)	B*07:02 (8.65)	B*15:01 (5.65)	B*35:01 (5.63)	B*53:01 (1.67)	
**RYGNFDILR**	7.09%	−	−	−	−	−	−	−	−	+	−	+	−	−	−	−	2
**TLDDLTWMDA**	40.60%	−	+	+	−	−	−	−	−	−	−	−	−	−	−	−	2
**LASYLHTFAY**	32.81%	+	−	−	−	−	−	−	+	−	−	−	−	+	+	−	4
**ASYLHTFAY**	58.51%	+	−	−	+	+	−	−	+	−	+	−	−	+	+	−	4
**WPAAGAWEL**	22.28%	−	−	−	−	−	−	−	−	−	−	−	+	−	+	+	7
**KVLPVGDEV**	40.60%	−	+	+	−	−	−	−	−	−	−	−	−	−	−	−	2
**VYKGAEVAHF**	26.18%	−	−	−	−	−	+	+	−	−	−	−	−	−	−	−	2
**Epitope set**	93.78%	2	2	2	1	1	1	1	2	1	1	1	1	2	3	1	22
**EPITOPES**	**Coverage**	**HLA Allele (genotypic frequency (%))**															**Total HLA Hits**
	**Class II**	DRB1*01:01 **(6.65)**	DRB1*03:01 **(10.47)**	DRB1*04:01(6.46)	DRB1*04:05 **(1.70)**	DRB1*07:01 **(10.71)**	DRB1*08:02(1.31)	DRB1*09:01 **(3.64)**	DRB1*11:01 **(6.06)**	DRB1*12:01(2.53)	DRB1*13:02 **(3.81)**	DRB1*15:01 **(10.82)**	DRB3*01:01 **(0.0)**	DRB3*02:02 **(0.0)**	DRB5*01:01 **(0.0)**	DRB5*01:01 **(0.0)**	
**IQNLKFSQSKQLKKL**	72.74%	+	+		−	+	+	+	+	+	+	+	−	+	+	+	11
**FSFSNLIQAVTRRFS**	81.81%	+	+	+	+	+	+	+	+	−	+	+	+	+	−	+	14
**KEAKFPILSANIKAK**	70.55%	+	−	+	+	+	+	+	+	+	+	+	−	−	+	+	11
**KLKTLNVNKIIALGH**	77.51%	+	+	+	−	+	+	−	+	−	+	+	−	+	+	+	12
**Epitope Set**	81.81%	4	3	3	2	4	4	3	4	2	4	4	1	3	3	4	48

+ Restricted: − Nonrestricted.

**Table 4:** List of B-cell epitopes selected for design of vaccine construct.

Protein	B-Cell Epitope	Start Position	Predicted Score	Antigenicity	Allergenicity/Toxicity	Server
MME (P08473)	QLKKLREKVDKDEWIS	522	0.93	1.20	NA/NT *	BCPred
	GYPDDIVSNDNKLNNE	481	0.97	0.74	NA/NT	
ANPEP (P15144)	PLFIHFRNNTNNWREI	728	0.98	1.05	NA/NT	BCPred
	NAIAQGGEEEWDFAWE	799	0.99	1.14	NA/NT	
NT5E (P21589)	VVVGGHSNTFLYTGNP	238	0.88	1.36	NA/NT	ABCpred
	NSSIPEDPSIKADINK	311	0.88	1.13	NA/NT	

B-cell epitopes were selected based on binding score (>0.9), high antigenicity, non-allergenicity and non-toxicity. * NA: Non-Allergenic, NT: Non-Toxic.

**Table 5:** Vaccine–receptor complex hydrogen bonds with different frames in MD simulation.

	TLR-2			TLR-3			TLR-4		
Frames	Number of Hydrogen Bonds	Receptor RMSD (Å)	Ligand RMSD (Å)	Number of Hydrogen Bonds	Receptor RMSD (Å)	Ligand RMSD (Å)	Number of Hydrogen Bonds	Receptor RMSD (Å)	Ligand RMSD (Å)
0 ns	14	0	0	20	0	0	18	0	0
10 ns	14	3.017	2.625	17	3.586	5.026	12	2.974	5.484
20 ns	13	3.333	6.035	13	3.605	6.001	11	3.597	6.478
30 ns	12	2.995	6.458	12	3.315	6.561	11	3.763	6.870
40 ns	14	2.869	6.311	13	3.530	6.939	13	4.298	6.583
50 ns	19	3.089	6.444	14	3.171	6.383	11	3.086	6.702
60 ns	20	3.014	6.648	13	3.165	6.402	12	3.258	6.881
70 ns	14	2.927	6.680	19	3.293	6.671	10	3.223	6.552
80 ns	18	3.028	6.872	17	3.478	6.392	11	3.400	6.840
90 ns	16	2.969	6.720	17	3.319	6.397	12	3.163	7.007
100 ns	15	3.072	6.742	26	3.322	6.537	15	3.017	6.907
**Average**	**15.36**	**2.75**	**5.59**	**16.45**	**3.07**	**5.75**	**12.36**	**3.07**	**6.03**

## Data Availability

The data presented in the study are included in this article and supplementary material. Further inquiries can be directed to the corresponding author.

## Supplementary Materials

The following supporting information can be downloaded at: https://www.mdpi.com/article/10.3390/vaccines10111850/s1, Figure S1: Schematic representation of vaccine construct developmental process; Figure S2: Predicted solubility of the vaccine construct; Table S1: Physicochemical properties of selected target proteins; Table S2: Predicted discontinuous B-cell epitope residues; Table S3: Docking scores of the vaccine construct with immune receptors; Table S4: The number of hydrogen bonds in the 100 ns simulation over 1000 time frames and corre-sponding receptor and ligand RMSD; Video S1: MD simulation analysis 1; Video S2: MD simulation analysis 2; Video S3: MD simulation analysis 3.
