# Supplementary material for: Designing of Peptide Based Multi-Epitope Vaccine Construct against Gallbladder Cancer Using Immunoinformatics and Computational Approaches
Source: Vaccines (Basel). 2022 Oct 31;10(11):1850. doi: 10.3390/vaccines10111850 (PMC9696659; doi:10.3390/vaccines10111850)
Supplement: Supplementary file 1 [file vaccines-10-01850-s001.zip › Supplementary Material.pdf]

Table S1: Physicochemical properties of selected target proteins

Proteins/Properties	NT5E (P21589)	ANPEP (P15144)	MME (P08473)
**Number of amino acids**	574	967	750
**Molecular weight**	63.75K Da	109.60 KDa	85.94 KDa
**Theoretical pI**	6.58	5.31	5.54
**Estimated half life**	30 hrs (Mammalian reticulocytes)<br>>20 hrs (Yeast)<br>>10 hrs (*E.coli*)	30 hrs (Mammalian reticulocytes)<br>>20 hrs (Yeast)<br>>10 hrs (*E.coli*)	30 hrs (Mammalian reticulocytes)<br>>20 hrs (Yeast)<br>>10 hrs (*E.coli*)
**Instability Index**	32.59	36.17	37.62
**Aliphatic Index**	94.39	84.51	83.37
**GRAVY**	-0.087	-0.317	-0.447
**Antigenicity**	0.5382	0.4971	0.4797
**Allergenicity**	Non-allergen	Non-allergen	Non-allergen

NT5E: 5′ -Nucleotidase isoform 2
ANPEP: Aminopeptidase N
MME: Membrane metallo-endopeptidase
GRAVY: Grand average of hydopathicity

Table S2: Predicted discontinuous B-cell epitope residues

Residues	Number of Residues	Score
A:D60, A:K61, A:E63, A:I65, A:S66, A:G67, A:P68, A:G69, A:P70, A:G71, A:G72, A:Y73, A:P74,A:D75, A:D76, A:I77, A:V78, A:S79, A:N80, A:D81, A:L84, A:N85	22	0.76
A:Y300, A:A301, A:A302, A:Y303, A:P305, A:A306, A:A307, A:G308, A:A309, A:W310, A:E311,A:L312, A:A313, A:A314, A:V328, A:Y329, A:K330, A:G331, A:A332, A:E333, A:V334, A:A335, A:H336, A:F337	24	0.73
A:H97, A:N100, A:N101, A:T102, A:N103, A:N104, A:W105, A:R106, A:E107, A:G108, A:P109,A:G110, A:E121, A:D124, A:F125, A:E128, A:G129, A:P130, A:G131, A:P132, A:G133, A:V134,A:V135, A:V136, A:G137, A:G138, A:H139, A:S140, A:N141, A:F143, A:L144, A:Y145, A:N148,A:P149, A:G150, A:P151, A:G152, A:P153, A:G154, A:N155, A:S156, A:S157, A:I158, A:P159,A:E160, A:D161, A:S182, A:S184, A:K185, A:L187, A:K188, A:K189, A:L190, A:G191, A:P192,A:G193, A:P194, A:G195, A:F196, A:S197	60	0.72
A:F220, A:P221, A:I222, A:L223, A:S224, A:A225, A:N226, A:I227, A:K228, A:A229, A:K230,A:G231, A:P234	13	0.71
A:G1, A:I2, A:I3, A:N4, A:T5, A:L6, A:Q7, A:Y9, A:Y10, A:C11, A:V13, A:R14, A:G15, A:G16, A:R17,A:C18, A:A19, A:V20, A:L21, A:S22, A:C23, A:E27, A:K44, A:K45, A:E46, A:A47, A:A49, A:K50,A:K53, A:G256, A:N257, A:F258, A:D259, A:I260, A:L261, A:R262, A:A263, A:A264, A:Y265,A:T266, A:L267, A:D268, A:D269, A:L270, A:T271, A:W272, A:M273, A:D274	48	0.67

Table S3: Docking scores of the vaccine construct with immune receptors

TLR Receptor	HDock Score	Hydrogen bonding residue (Lig-Plot)
**TLR-2**	-344.38	Lys8, Arg207, Ile30, Asn200, Tyr255, Asp259, Glu333
**TLR-3**	-345.38	Asp268, Arg262, His336, Asp259, Asn85, Glu87, Asp322, Arg12, Arg14
**TLR4**	-324.47	Ala299, Arg207, Ser199, Gln29, Lys32, Lys8

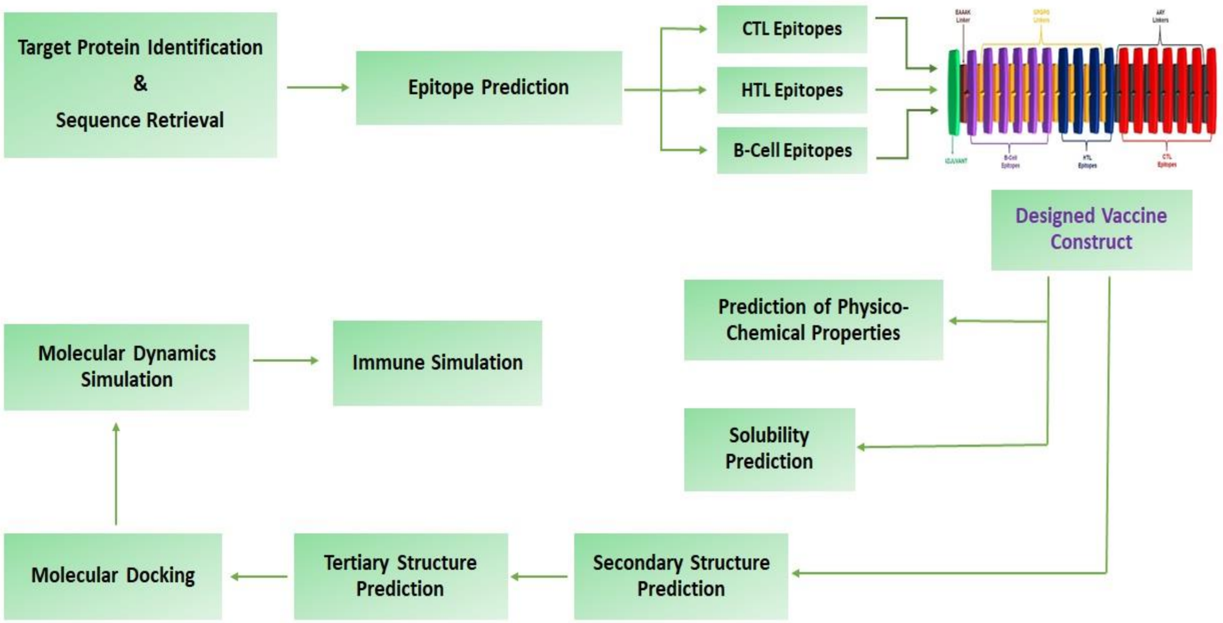

Figure S1: Schematic representation of vaccine construct developmental process

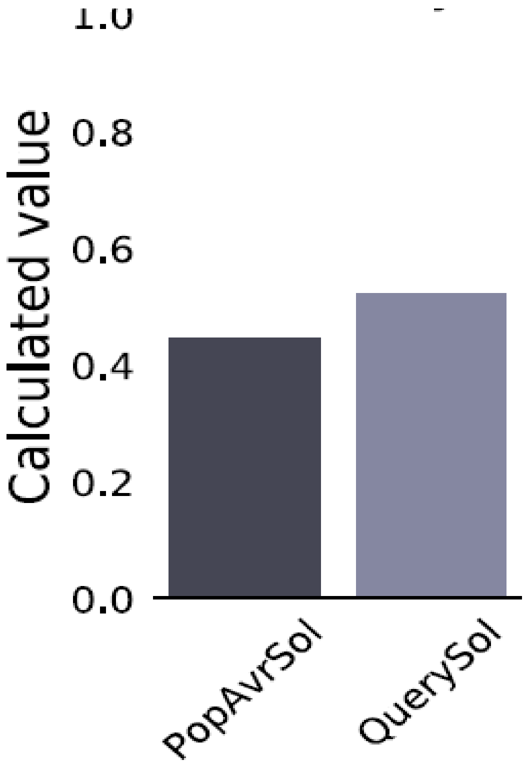

Figure S2: Predicted solubility of the vaccine construct

Query Sol represents the predicted scaled solubility of the vaccine construct. The population average for the experimental dataset (PopAvrSol) is 0.45; therefore any scaled solubility value > 0.45 is predicted to have a higher solubility than the average soluble E.coli protein from the experimental solubility dataset.
